# Quality Assessment of Clinical Practice Guidelines Developed by Professional Societies in Turkey

**DOI:** 10.1371/journal.pone.0156483

**Published:** 2016-06-13

**Authors:** Ilknur Yaşar, Rabia Kahveci, Aylin Baydar Artantaş, Duygu Ayhan Başer, Fatma Gökşin Cihan, Irfan Şencan, Esra Meltem Koç, Adem Özkara

**Affiliations:** 1 Department of Family Medicine, Ankara Yenimahalle Public Health Center, Ankara, Turkey; 2 Department of Family Medicine, Ankara Numune Training and Research Hospital, Ankara, Turkey; 3 Department of Family Medicine, Ankara Training and Research Hospital, Ankara, Turkey; 4 Department of Kocaeli Peliublic Health Directorate, Health of Child, Adollesence,Woman Reproduction, Koc, Turkey; 5 Department of Family Medicine, Konya Necmettin Erbakan University, Konya, Turkey; 6 Department of Family Medicine, Ankara Mamak Public Health Center, Ankara, Turkey; University of South Australia, AUSTRALIA

## Abstract

**Background:**

Clinical practice guidelines (CPGs) are systematically developed statements to assist practitioner and patient decisions about appropriate healthcare for specific clinical circumstances. There is a limited number of studies on guidelines in Turkey. The quality of Ministry of Health guidelines have formerly been assessed whereas there is no information on the other guidelines developed in the country.

**Aim:**

This study aims to assess the quality of CPGs that are developed by professional societies that work for the health sector in Turkey, and compare the findings with international guidelines.

**Methodology:**

Professional societies that work for the health sector were determined by using the data obtained from the Ministry of Internal Affairs. Inclusion and exclusion criteria were defined for selecting the CPGs. Guidelines containing recommendations about disease management to the doctors, accessible online, developed within the past 5 years, citing references for recommendations, about the diseases over 1% prevalence according to the “Statistical Yearbook of Turkey 2012” were included in the study. The quality of CPGs were assessed with the AGREE II instrument, which is an internationally recognized tool for this purpose. Four independent reviewers, who did not participate in the development of the selected guidelines and were trained in CPG appraisal, used the AGREE instrument for assessment of the selected guidelines.

**Findings:**

47 professional societies were defined which provided access to CPGs in their websites; 3 of them were only open to members so these could not be reached. 8 CPGs from 7 societies were selected from a total of 401 CPGs from 44 societies. The mean scores of the domains of the guidelines which were assessed by the AGREE II tool were; Scope and purpose: 64%, stakeholder involvement: 37.9%, rigour of development: 35.3%, clarity and presentation: 77.9%, applicability: 49.0% and editorial independence: 46.0%.

**Conclusion:**

This is the first study in Turkey regarding quality appraisal of guidelines developed by the local professional societies. It adds to the limited amount of information in the literature that comes from Turkey as well as other developing countries.

## Introduction

In the last 20 years, clinical practice guidelines (CPGs) have progressively become a popular tool for the synthesis of correct and updated information [1]. This is because CPGs are believed to increase the quality, appropriateness and cost effectiveness of the health sector [2,3]. In 2011, The Institute of Medicine (IOM) defined Clinical Practice Guidelines as “statements that include recommendations intended to optimize patient care that are informed by a systematic review of evidence and an assessment of the benefits and harms of alternative care options” [4].

In clinical practice, publication of widely-used guidelines of different qualities [5,6] has made it an obligation to differentiate high-quality guidelines from low-quality ones. Various assessment tools have been developed in many countries of America and Europe to evaluate the quality of guidelines [7]. In the study of Siering et al., in which they investigated the assessment tools developed between 1995–2011, 40 different guideline assessment tools were found. That study determined 13 principle quality tools that must be taken into account during assessment of guidelines: Information retrieval, Evaluation of evidence, Consideration of different perspectives, Formulation of recommendations, Transferability, Presentation of guideline content, Alternatives, Reliability, Scope, Independence, Clarity and presentation, Updating, Dissemination, Implementation, and Evaluation. According to this study, the AGREE II Instrument developed by the AGREE collaboration in 2009 and the German tool, DELBI, fulfilled 100% of these 13 principle quality measures [8]. AGREE II has been used as the assessment tool in many studies evaluating the quality of guidelines [9,10,11]. The possible reason for this choice is because AGREE is an international and widely accepted tool whose validity has been approved, is easy to use and is transparent [12]. In the studies performed by Vyalen et al. which analyzed guideline assessment tools, AGREE was found to be the sole valid tool [13]. Presently, Turkey does not have its own guideline assessment tool. Artantas et al., who performed the first guideline quality assessment study in Turkey, translated the original AGREE Instrument into Turkish and assessed primary care guidelines which were published by the Ministry of Health (MoH) [14,15,16]. Although the study provided a general opinion of quality of MoH guidelines targeting primary care, it was not possible to generalize the findings to all CPGs in the country. CPGs are developed either by MoH or Professional societies in Turkey and these societies are known to develop more comprehensive guidance for different levels of care. On the other hand, no evidence is available to show whether they have better uptake or have higher quality. The objective of the present study is to assess the quality of the guidelines introduced by professional societies working in healthcare in Turkey, compare it with MoH guidelines and also with the findings of international guidelines.

## Materials And Methods

### Selection of clinical practice guidelines

There were 959 professional societies working in the field of health and serving as non-governmental organizations (NGOs) under the Turkish Ministry of Internal Affairs and each NGO was manually searched on the internet. Among the NGOs with an internet site, those with a CPG were found to be 47, presenting a total of 402 guidelines. The guidelines were listed according to whether they were presented in Turkish or English, and according to their originality, adaptation and translational nature. Taking into consideration the below inclusion and exclusion criteria designed by the researchers, a total of 8 clinical CPGs presented by 7 NGOs were included in our study [Table 1].

**Table 1:** Guidelines included in the study according to the inclusion criteria.

Guidelines	Professional Societies	Access link for the guideline
	Society for Pediatric Nephrology	http://cocuknefroloji.org.tr
Turkish Enuresis Guideline (2010) [18,19,20]	Society for Pediatric Urology	http://www.peduro.org.tr
	Turkish Society of Pediatric Surgery	http://www.tccd.org.tr
Guideline on the diagnosis and treatment of COPD (2010) [21]	Turkish Thoracic Society	http://www.toraks.org.tr
Guideline on the Diagnosis and Treatment of Asthma (2010) [21]		
Guideline on the Diagnosis and Treatment of Erythrocyte Diseases and Hemoglobin Disorders (2011) [22]	Turkish Society of Hematology	http://www.thd.org.tr
Guideline on the Diagnosis and Treatment of Thyroid Diseases (2012) [23]		
Obesity, Dyslipidemia, Hypertension Diagnosis and Treatment Guideline for Physicians (2012) [23]	The Society of Endocrinology and Metabolism of Turkey	http://www.turkendokrin.org
Guideline on the Diagnosis, Treatment and Follow-up of Diabetes Mellitus and its Complications (2013) [23]		
Guideline on the Diagnosis and Treatment of Headache (2010) [24]	Turkish Society of Neurology	http://www.noroloji.org.tr

**Inclusion criteria**
1. Guidelines developed for doctors and containing recommendations related to prevention, diagnosis and rehabilitation of diseases
2. Guidelines developed in Turkey and in Turkish language
3. Guidelines related to disease/health problems with a prevalence of >1% among individuals older than 15 years, according to “Statistical Yearbook of Turkey 2012” [17]
4. Guidelines developed and published within the last 5 years
5. Guidelines completely accessible online

**Exclusion criteria**
1. Short texts containing technical information
2. Educational guidelines for medical experts
3. Guidelines for patients
4. Technical guidelines related to assessments and diagnostic tools
5. Health guidelines of government developed for health control
6. Translated guidelines
7. Guidelines developed for professionals other than medical doctors (Nurses, dentists etc.)

### The AGREE II Instrument

The AGREE II Instrument, which was used for the assessment of guidelines, is an updated version of the original AGREE Instrument developed in 2003 by the AGREE Collaboration. Similar to the original AGREE Instrument, the AGREE II Instrument consists of 23 items organized within six domains and 2 overall assessment items. Each domain captures a specific aspect of guideline quality. **Domain 1. Scope and Purpose-** overall aim of the guideline, specific health problems, and target group. **Domain 2. Stakeholder Involvement-** extent to which appropriate stakeholers were involved in developing the guideline and represents the views of its intended users. **Domain 3. Rigour of Development-** process of gathering and summarizing the evidence, methods used to develop recommendations, and updating them. **Domain 4. Clarity of Presentation-** language, structure and format of guideline. **Domain 5. Applicability-**potential barriers and facilitators to implementation, strategies to improve uptake, resources needed to implement the guideline. **Domain 6. Editorial Independence-** biases due to competing interests. Overall assessment includes rating the overall quality of the guideline and whether the guideline would be recommended for use in practice. Each domain and the two overall assessment items of AGREE II are rated over a scale of 7 points (from 1-definitely disagree to 7- definitely agree). Domain scores are calculated by summing up all the scores of items in the domain and by scaling the total as a percentage of the maximum possible score for that specific domain. Domain points are useful for comparison of guidelines and provide information on whether a guideline might be recommended. On the other hand the tool does not specify a certain threshold to differentiate a high quality guideline from a low quality guideline. Here, users are expected to decide on the quality of the guidelines and also on the recommendation for clinical use [18].

In general, the purpose of AGREE II is to assess the quality of guidelines, to provide a methodological strategy for the development of guidelines and to inform what information and how the information ought to be reported in guidelines [18].

### Translation of The AGREE II Instrument to Turkish

The Turkish version of the AGREE II Instrument was developed by the Turkish Evidence Based Medicine Society [16], following the translation process addressed by the AGREE Collaboration, and was used in this study to assess the specified clinical practice guidelines.

### Assessment of Clinical Practice Guidelines

The selected guidelines were assessed by 4 reviewers who were well acquainted about the use of the AGREE II Instrument, and had no role in the development and other processes of the selected guidelines. The findings were later compared with previous findings of Artantaş [15]regarding quality of the CPGs published by Turkish MoH. The findings were also compared with findings of Allonso-Coello who evaluated guidelines published between 1980–2007 [9].

## Results

Among the 402 guidelines, 8 guidelines fulfilling the specified criteria were included in the study [Table 1]. The scores achieved by the included guidelines in 6 quality domains are shown in Table 2.

**Table 2:** Scores achieved by guidelines in each AGREE II domain.

	Scope and Purpose (%)	Stakeholder Involvement (%)	Rigour of Development (%)	Clarity of Presentation (%)	Applicability (%)	Editorial Independence (%)
Guideline on the Diagnosis and Treatment of Asthma	79.1	58.3	45.3	84.7	63.5	35.4
Guideline on the Diagnosis and Treatment of COPD	70.8	51.3	45.8	76.3	54.1	27
Guideline on the Diagnosis and Treatment of Headache	41.6	25	13	59.7	20.8	10.4
Turkish Enuresis Guideline	84.7	54.1	45.3	81.9	53.1	16.6
Guideline on the Diagnosis and Treatment of Thyroid Diseases	50	26.3	24.4	77.7	44.7	79.1
Guideline on the Diagnosis, Treatment and Follow-up of Diabetes Mellitus and its Complications	79.1	45.8	63	93	63.5	91.6
Guideline on the Diagnosis and Treatment of Erythrocyte Diseases and Hemoglobin Disorders	50	23.6	22.3	68	44.7	27
Obesity, Dyslipidemia and Hypertension Diagnosis and Treatment Guideline for Physician	56.9	19.4	23.4	81.9	47.9	81.2
**Average scores achieved in each domain by the guidelines**	**64**	**37.9**	**35.3**	**77.9**	**49.3**	**46.3**

### Findings on domain scores

The domain scores between the eight guidelines differed significantly: Scope and Purpose (41.6–84.7, with average 64); Stakeholder Involvement (19.4–58.3, average 37.9); Rigour of Development (13–63, average 35.3); Clarity of Presentation (59.7–93, average 77.9); Applicability (20.8–63.5, average 49.3) and Editorial Independence (10.4–91.6, average 46.3). While Rigour of Development received the lowest average scores, Clarity of Presentation received the highest.

The domain scores for each guideline would also significantly differ, which could be followed from Table 2. The findings suggest that while one CPG could receive low scores in certain domains it might still end up with high scores in others and this certainly influences reviewers’ recommendations.

“Guideline on the Diagnosis and Treatment of Headache” achieved the lowest score as 10,4% in the Editorial Independence domain and 13% in Rigour of Development domain. The highest quality score was achieved by the guideline “Guidelines on the Diagnosis, Treatment and Follow-up of Diabetes Mellitus and its Complications” in the domains Clarity of Presentation (93%) and Editorial Independence (91.6%). “Guidelines on the Diagnosis and Treatment of Headache” was also the guideline to achieve the lowest score in five of six domains.

Clarity of Presentation was the domain to achieve the highest score with an average of 77.9% while Rigour of Development was the domain to achieve the lowest score with an average of 35.3%.

### Findings on overall assessment and recommendations

Table 3 shows the overall quality scores and views of the reviewers related to recommendation of the guidelines. Overall quality scores ranged from 2 to 5.25 over a maximum 7. While the “Guideline on the Diagnosis, Treatment and Follow-up of Diabetes Mellitus and its Complications” achieved the highest score, “Guidelines on the Diagnosis and Treatment of Headache” achieved the lowest.

**Table 3:** Overall assessment of the reviewers regarding recommendation of guidelines and average overall quality score.

	Reviewer 1	Reviewer 2	Reviewer 3	Reviewer 4	Average overall quality score
Guideline on the Diagnosis and Treatment of Asthma	Yes, with modifications	Yes, with modifications	Yes, with modifications	Yes, with modifications	4.5
Guideline on the Diagnosis and Treatment of COPD	Yes, with modifications	Yes, with modifications	Yes,	Yes,	4,5
Guideline on the Diagnosis and Treatment of Headache	No	No	Yes, with modifications	No	2
Turkish Enuresis Guideline	Yes, with modifications	Yes, with modifications	Yes,	Yes, with modifications	4,75
Guideline on the Diagnosis and Treatment of Thyroid Diseases	No	Yes, with modifications	Yes,	Yes, with modifications	4
Guideline on the Diagnosis, Treatment and Follow-up of Diabetes Mellitus and its Complications	Yes, with modifications	Yes, with modifications	Yes,	Yes,	5,25
Guideline on the Diagnosis and Treatment of Erythrocyte Diseases and Hemoglobin Disorders	No	Yes, with modifications	Yes, with modifications	Yes, with modifications	3,5
Obesity, Dyslipidemia, Hypertension Diagnosis and Treatment Guideline for Physicians	No	Yes, with modifications	Yes, with modifications	Yes, with modifications	4,25

Reviewers’ recommendations were in complete agreement in only one of the guidelines. There were differences in comments in others. “Guideline on the Diagnosis and Treatment of Headache” -which achieved low scores in all domains in general- was not recommended for use by three reviewers while one reviewer recommended its use only after making changes in the guideline. “Guideline on the diagnosis and treatment of Chronic Obstructive Pulmonary Disease (COPD)” and “Guideline on the Diagnosis, Treatment and Follow-up of Diabetes Mellitus and its Complications” were recommended by two reviewers in its present state.

When the decision of each of the reviewers was analyzed separately, most of the reviewers found the guidelines recommendable only after sorting out their shortcomings.

An interesting finding was to see that the reviewer who was most experienced in CPG development and quality appraisal (Reviewer 1) did not recommend any CPG without modification, recommended four of them with modification and refused to recommend the rest. On the other hand, the least experienced one (Reviewer 3) recommended four guidelines with no modification and recommended the others with modifications.

### Comparison with other quality assessment studies

Table 4 summarizes domain scores from this study and compares it to findings from Artantaş [15] and Allonso-Coello [9]. Allonso-Coello assessed a broad range of CPGs on different topics in almost three decades and provides an international sight, while Artantaş shares the only available assessment related to CPG quality in Turkey and makes national comparison possible.

**Table 4:** Average scores achieved by guidelines in AGREE II domains and comparison with average scores of Moh Guidelines and international guidelines.

	Scope and Purpose (%)	Stakeholder Involvement (%)	Rigour of Development (%)	Clarity of Presentation (%)	Applicability (%)	Editorial Independence (%)
Average scores of NGO guidelines	64	37.9	35.3	77.9	49.3	46.3
Average scores of MoH guidelines	87.9	62.2	51.2	66.4	57.2	54.5
AGREE International average scores[9].	64	35	43	60	22	30

In both MoH and NGO guidelines lowest scores are observed on Rigour of Development domain. International scores, although not lowest, also suggest that Rigour of Development is a domain that has relatively low scores. Both MoH guidelines and international guidelines have highest scores on Scope and Purpose domain. International guidelines received lowest scores in Applicability.

## Discussion

The selection of guidelines in this study was done according to inclusion criteria determined by the researchers and among a total of 402 guidelines, 8 were suitable for inclusion. One of the limitations of this study is that it involves a limited number of local guidelines and its results can neither be generalized to other clinical practice guidelines that are not included nor to the guidelines developed by the public institutions. This is the major limitation that makes it hard for us to comment on general quality of CPGs in the country. As there were almost 1000 sites searched and the retrieved CPGs were over 400 we had to limit our inclusion criteria and over 1% prevalence was decided to be a criteria to have a sound method. We also thought that a future study including the rest could also provide additional information about the CPGs targeting rare conditions. The number 8 we finally received was less than we expected, and made it harder to make general assumptions. A second limitation is the difficulty in getting access to guidelines. Since there is no national clearinghouse in Turkey that accomodates all clinical practice guidelines, manual screening of web sites of almost 1000 societies was necessary in order to access the guidelines. During this extensive work, although double-checked by two researchers, there is always a possibility that we have missed a guideline that could have been potentially included in the study. Lastly, differentiation of whether the accessible guidelines were de novo, translated, or adapted was not always possible. Some guidelines did not mention this issue and some de novo guidelines might actually be adaptation by nature. This, on the other hand, does not mean that their quality assessment is not valuable. As these are the available Turkish CPGs provided to the doctors, it is worth assessing their quality even if they are adaptation or translation by nature. In spite of the limitations, the major strength of our study is that it is the first study to assess guidelines presented by non-governmental organizations working in the field of health in Turkey, and thus, it gives us an idea about the quality of selected guidelines of our country, and makes important contribution to international literature in this field, especially from countries where there is scarce information.

Our study has determined three important findings. Firstly, there are only 8 guidelines related to 19 disease/health problems with a prevalence of >1% in individuals older than 15 years. In the study by Artantas et al., 14 guidelines presented by The Ministry of Health which were related to the 10 most common diseases were assessed [15]. Although that study concluded that guideline quality is not at an acceptable level, the presence of guidelines related to problems most frequently seen in the population is significant. Our study demonstrated that professional societies of Turkey are inefficient in developing guidelines. Second finding is that it is not clear what care level of physicians these guidelines are aimed at. There is no guideline development attempt of societies of primary care, while other professional societies do not separately take into account approaches at other care levels and also do not mention their target care level. The cause of insufficiency of guidelines relating to the most prevalent diseases in Turkey may be a separate investigation topic of interest. A third finding is that no methodologic approaches are built up for guideline development by either public or non-governmental organizations. Only one non-governmental organization has a guideline development directive available. No activity besides that of the Society of Evidence Based Medicine is present relating to quality assessment.

Quality assessment of the selected guidelines with AGREE II reveals that Clarity of Presentation along with Scope and Purpose were the most successful domains while Stakeholder Involvement, Rigour of Development and Editorial Independence were the least successful domains. The successful and unsuccessful domains are similar to previous studies which assessed guidelines aimed at primary care physicians. The low scores in Rigour of Development suggests the need for further training in EBM skills, because this domain is basically related to how we work with sound evidence to build up our recommendations. The low scores in Stakeholder Involvement and Editorial Independence, on the other hand must be seen as a different aspect of the guideline development and use. The involvement of relevant stakeholders and its declaration will ensure different views to be taken into account, improve acceptability and applicability. Editorial independence is important for accountability. Three CPGs in this study are funded by pharmaceutical companies, three declared no funding and the other two has no declaration. The declaration of funding should not be considered as enough by itself, but needs further clarification how this might have affected the content. Declaration of conflicts will further give the opportunity to the authors to manage these conflicts. Editorial independence has also been considered as an important issue in other guidelines and has not been observed to enhance over years [9].

The lack of threshold value of points calculated for quality domains in the AGREE Instrument has encouraged investigators to find and use a threshold value, and in some studies [19,20], investigators have used self-defined threshold values to determine the quality of guidelines. We did not specify any threshold value for domain points in our study. However, when the points achieved by the investigated guidelines were compared with international average, “Guidelines on the Diagnosis and Treatment of Headache” was below average in all the domains. Thus, 3 of the 4 reviewers have interpreted this guideline as “I do not recommend its use”. The guideline achieving scores above average in all domains were “Guidelines on the Diagnosis and Treatment of Asthma” and “Guidelines on the Diagnosis, Treatment and Follow-up of Diabetes Mellitus and its Complications”. In the overall quality score which was not present in the original AGREE instrument but was added in AGREE II, “Guidelines on the Diagnosis, Treatment and Follow-up of Diabetes Mellitus and its Complications” achieved the highest scores. This guideline is widely followed, at a rate of 71.3±21%, according to the ADMIRE Study performed by Satman et al. [21]. This shows that the use of this guideline, which achieved the highest overall quality score in our study, provides great success in the management of diabetes which is a chronic disease with a very high prevalence in our country.

In this study, we tried to compare our results with international findings and previous Turkish studies. There was only one available Turkish study, which made it hard for us to compare and to make a general assumption on Turkish guidelines. Our comparison with Allonso-Coelho’s findings also has a limitation, due to differences in time of publication, topic of interest and settings. Further studies on specific topics and their comparison with international guidelines on the same topics might be useful for guideline developers. We did not do this, as we did not focus our study on management of certain conditions and this was beyond the scope of this study.

Guidelines of larger organizations engaged specifically in development of guidelines also have shortcomings similar to the guidelines we investigated [11,22]. The study by Oxman et al. in 2007 showed that the recommendations of WHO are primarily based on expert opinion and that very rarely do they consist of systematic evidence based methods. These results have encouraged WHO to form Guideline Investigation Committee [23]. Sinclair et al., in 2013, carried out a study assessing the quality of guidelines developed by WHO before and after the formation of Guideline Investigation Committee with the AGREE II Instrument and compared them. It was found that the quality of guidelines increased after the formation of Guideline Investigation Committee in terms of all domains, especially in Rigour of Development and Editorial Independence domains, and new standards determined by the committee were found to increase average scores of all domains to a level above AGREE II international average score [11]. This shows that formation of development and audit units by guideline-developing organizations and development of guidelines with the correct and standard procedures are the most important factors that increase quality. Besides, use of assessment tools such as AGREE II during guideline development phase allows the guideline-developing team to follow the process step-by-step. Another point that was noticed during assessment was that no information regarding Stakeholder Involvement and Editorial Independence domains were stated in most of the guidelines. This resulted in guidelines receiving very low points in these domains. Although some guidelines are known to be developed according to these criteria, this information has not been shared in the guideline document; as a result, such guidelines have been accepted as failing to meet required criteria and this has led to reception of low scores.

## Conclusion

There are very few guidelines dealing with the most prevalent diseases in Turkey and no repository is present for the physicians to get easy access to these guidelines. When the quality of guidelines are evaluated methodologically, significant shortcomings, especially in the Rigour of Development and Editorial Independence domains, are observed. Similar results obtained for guidelines developed by both the Ministry of Health and non-governmental organizations may pave a way for steps to be taken to improve the quality of guidelines. This is the first study that explores quality of guidelines developed by professional societies in Turkey, and thus we believe that it will be a valuable reference source for investigators interested in this topic. Besides, this study may contribute to international literature regarding the quality of guidelines in developing countries and may allow for international comparisons.
